# Exploring the Diversity and Distribution of Neotropical Avian Malaria Parasites – A Molecular Survey from Southeast Brazil

**DOI:** 10.1371/journal.pone.0057770

**Published:** 2013-03-01

**Authors:** Gustavo A. Lacorte, Gabriel M. F. Félix, Rafael R. B. Pinheiro, Anderson V. Chaves, Gilberto Almeida-Neto, Frederico S. Neves, Lemuel O. Leite, Fabrício R. Santos, Érika M. Braga

**Affiliations:** 1 Departamento de Parasitologia, Universidade Federal de Minas Gerais, Belo Horizonte, Minas Gerais State, Brazil; 2 Departamento de Biologia Geral, Universidade Federal de Minas Gerais, Belo Horizonte, Minas Gerais State, Brazil; 3 Centro de Ciências Biológicas e da Saúde, Universidade Estadual de Montes Claros, Montes Claros, Minas Gerais State, Brazil; Université Pierre et Marie Curie, France

## Abstract

Southeast Brazil is a neotropical region composed of a mosaic of different tropical habitats and mountain chains, which allowed for the formation of bird-rich communities with distinct ecological niches. Although this region has the potential to harbor a remarkable variety of avian parasites, there is a lack of information about the diversity of malarial parasites. We used molecular approaches to characterize the lineage diversity of *Plasmodium* and *Haemoproteus* in bird communities from three different habitats in southeast Brazil based on the prevalence, richness and composition of lineages. We observed an overall prevalence of 35.3%, with a local prevalence ranging from 17.2% to 54.8%. Moreover, no significant association between prevalence and habitat type could be verified (p>0.05). We identified 89 *Plasmodium* and 22 *Haemoproteus* lineages, with 86% of them described for the first time here, including an unusual infection of a non-columbiform host by a *Haemoproteus* (*Haemoproteus*) parasite. The composition analyses of the parasite communities showed that the lineage composition from Brazilian savannah and tropical dry forest was similar, but it was different from the lineage composition of Atlantic rainforest, reflecting the greater likeness of the former habitats with respect to seasonality and forest density. No significant effects of habitat type on lineage richness were observed based on GLM analyses. We also found that sites whose samples had a greater diversity of bird species showed a greater diversity of parasite lineages, providing evidence that areas with high bird richness also have high parasite richness. Our findings point to the importance of the neotropical region (southeast Brazil) as a major reservoir of new haemosporidian lineages.

## Introduction

Avian malaria is a vector-borne disease caused by related parasites of two genera, *Plasmodium* and *Haemoproteus* (Phylum *Apicomplexa*, class Haemosporida), which are globally distributed occurring in most bird species [1]–[2]. Although the symptoms of avian malarial infection are generally mild, some evidence has shown that these parasites can exert an important selective pressure on their hosts through effects on survival, reproductive success, behavior and community structure [3]–[8].

During the past few decades, the application of DNA sequencing and the definition of cytochrome *b* (cyt-*b*) as a molecular marker to identify parasite lineages has revealed that avian malarial lineages are as diverse as their hosts, providing support to various studies on diversity, distribution, migration patterns and host specificity. This has attracted the attention of ecologists and evolutionary biologists to the utility of malarial infections as a model to study host-parasite systems [9]–[14].

Recently, several studies have been conducted to elucidate the relationships of malarial parasites and their hosts at the community level [11], [15]–[17]. These studies have shown that the prevalence of infection and the richness of parasites in a community is a result of complex interactions between biotic and abiotic components. The abiotic factors, mainly climatic conditions and habitat characteristics, can impact host (vertebrate and vector) diversity and abundance, resulting in changes in parasite transmission [18]–[19], whereas biotic factors associated with the hosts, such as behavior, age, sex, migration patterns and immunity strategies can also affect the success of infection and transmission [11], [20].

Brazil is a tropical country exhibiting a remarkable diversity of ecosystems that harbors one of the richest avifauna populations in the world [21]. Indeed, southeast Brazil deserves special attention because this region includes a mosaic of four different biomes, with a range of phyto-physiognomies and contact zones, allowing for the formation of bird communities with distinct ecological profiles [22]. In addition, the current models used to explain the temporal variation in parasite prevalence and parasitemia were proposed for temperate environments [2], [23]. These models are most likely not applicable to parasite communities from southeast Brazil due to some unusual features such as (1) Brazilian avifauna is mostly composed of non-migratory species [24]; (2) variation in vector densities is not observed in most of the habitats; and (3) relapse events are not a common phenomenon in tropical bird species [2].

Although southeast Brazil has the potential to harbor a remarkably rich population of avian malaria parasites due the diversity of habitats and host species, there is a lack of information about the diversity of malaria parasites in tropical bird communities [25]. Therefore, our primary goals were to characterize the lineage diversity of *Plasmodium* and *Haemoproteus* in bird communities from southeast Brazil, to infer the phylogenetic relationships of these parasites and to identify potential differences in prevalence, richness and composition of lineages among the habitats. The characterization of the malarial parasite communities presented here is essential for further studies that will address host-parasite systems in neotropical environments.

## Results

Among 1,545 bird samples representing 194 species screened for *Plasmodium* or *Haemoproteus* infection, we detected an overall prevalence of 35.3% (545 positive samples) comprising 132 infected species (Table 1). The prevalence varied significantly among the sites, ranging from 17.2% to 54.8%, and was also quite different among the bird species; for example, some species, such as *Thamnophilus ambiguus, Turdus leucomelas* and *Conopophaga lineata* (71%, 67%, and 65%, respectively), had high prevalence values, and 62 bird species had no detectable infection.

**Table 1:** Summary information from each sampling site, including infection data.

Habitat	Site	N	Infected samples	Bird richness	Lineage richness
***Atlantic rainforest*** ***(AF)***	Aracruz (ARA)	84	27	26	13
	Nova Lima (NOV)	164	57	45	24
	Sooretama (SOO)	86	13	21	8
	Caratinga (CAR)	126	53	33	23
***Brazilian savannah*** ***(BS)***	Felixlândia (FEL)	175	96	40	22
	Bocaiúva (BOC)	295	51	60	22
	Brasilândia (BRA)	133	62	55	28
***Tropical dry forest*** ***(DF)***	Salto da Divisa (SAL)	196	97	53	33
	Manga (MAN)	120	27	41	11
	Jequitinhonha (JEQ)	166	62	48	22
	Total	1545	545	194	110

Unfortunately, some samples were not successfully amplified nor cyt-b sequenced. We recovered sequences from 78% of the positive samples; 358 of these were positive for *Plasmodium* (84%), and only 70 were positive for *Haemoproteus*. Based on these sequences, we found 110 different cyt-*b* lineages, with 89 *Plasmodium* and 21 *Haemoproteus* lineages (Table S1), and most of these were recorded for the first time: 77 *Plasmodium* and 19 *Haemoproteus* lineages. Moreover, we detected 16 mixed infections: 13 cases of double *Plasmodium* infection, two cases of double *Haemoproteus* infection, and one case of *Plasmodium*/*Haemoproteus* infection. The lineage richness varied significantly among the sites, ranging from 8 to 28 lineages.

The host range of *Plasmodium* lineages varied from a single species to 11 host species, whereas *Haemoproteus* lineages presented a narrower range, varying from 1 to 7 host species (Fig. S1). Moreover, the most frequent *Plasmodium* and *Haemoproteus* lineages (PADOM09 and ELALB01, respectively) were also the most widespread among the sites and host species. The observed number of lineages harbored by a bird species ranged from a single lineage to 18 distinct lineages. However, the data from single lineage infections should be considered with caution because only five out of the 66 lineages observed in a single host species presented more than five records (Table S1).

**Figure 1:**
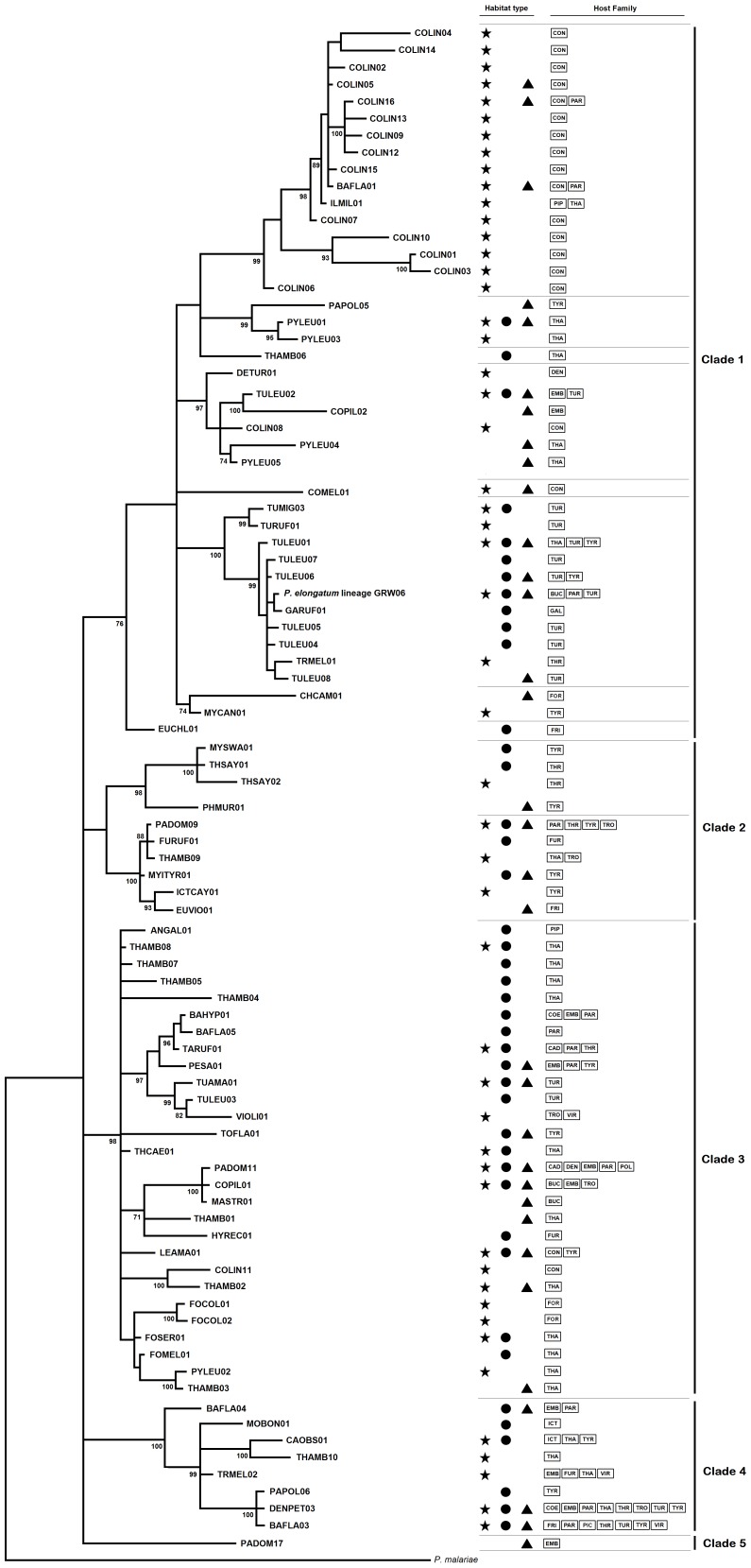
Phylogenetic relationships of the *Plasmodium* cyt-b lineages. *Plasmodium malariae* was used as outgroup. Numbers located near of the branches indicate Bayesian probability values. Symbols depict the different habitats (star: Atlantic rainforest; circle: Brazilian savannah; triangle: seasonally dry forest). Family birds are coded (BUC = Bucconidae; CAD = Cardinalidae; COE = Coerebidae; CON = Conopophagidae; DEN = Dendrocolaptidae; EMB = Emberezidae; FOR = Formicaridae; FRI = Fringilidae; FUR = Furnaridae; GAL = Galbulidae; ICT = Icteridae; PAR = Parulidae; PIC = Picidae; PIP = Pipridae; POL = Polioptidae; THA = Thamnophilidae; THR = Thraupidae; TRO = Troglodytidae; TUR = Turdidade; TYR = Tyranidae; VIR = Vireonidae).

**Figure 2:**
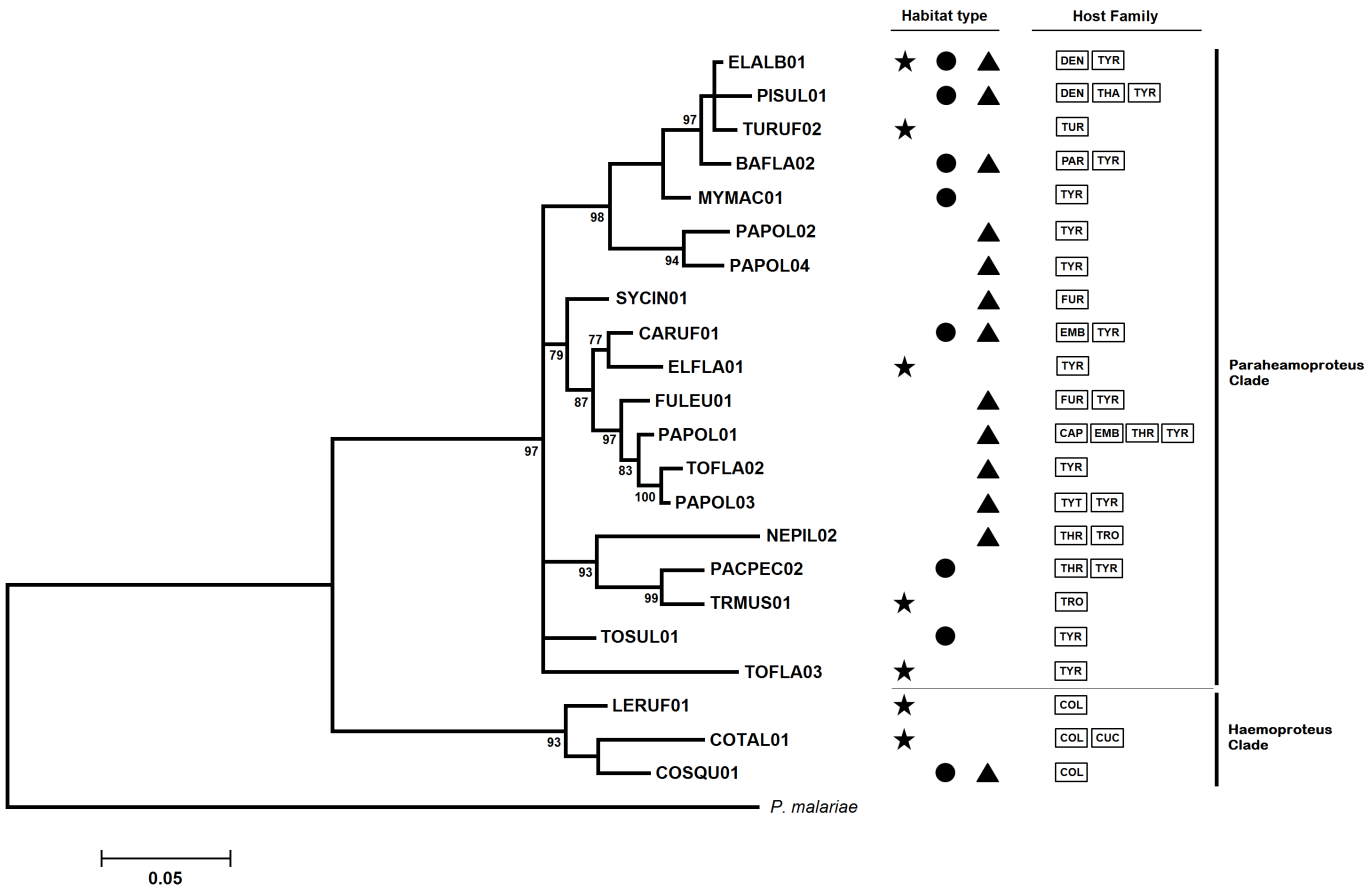
Phylogenetic relationships of the *Haemoproteus* cyt-b lineages. *Plasmodium malariae* was used as outgroup. Numbers located near of the branches indicate Bayesian probability values. Symbols depict the different habitats (star: Atlantic rainforest; circle: Brazilian savannah; triangle: seasonally dry forest). Family birds are coded as shown in figure 2.

The phylogenetic analysis of the *Plasmodium* cyt-b sequences revealed five highly supported clades and several subclades (Fig. 1); however, the relationships among these clades could not be estimated due to the poor resolution of the deep tree branches. The average genetic distances within the clades ranged from 3.4% to 6.0%, whereas the average distances among the clades varied from 6.3% to 8.7%. Only a subclade of Clade 1 presented an association with a habitat type, these lineages were mostly detected in birds from Atlantic rainforest sites. The phylogenetic tree of *Haemoproteus* lineages revealed two highly supported clades corresponding to the commonly reported “*Haemoproteus*” and “*Parahaemoproteus*” subclades (Fig. 2). Although most of the lineages of the “*Haemoproteus*” clade infected bird species of the Columbidae, we found two unusual infections of a Cuculidae species, *Coccyzus melacoryphus*, by a lineage (COTAL01) belonging to this clade (Table S1). In addition, we observed that 78% of *Haemoproteus* lineages were found exclusively in a single habitat type.

**Figure 3:**
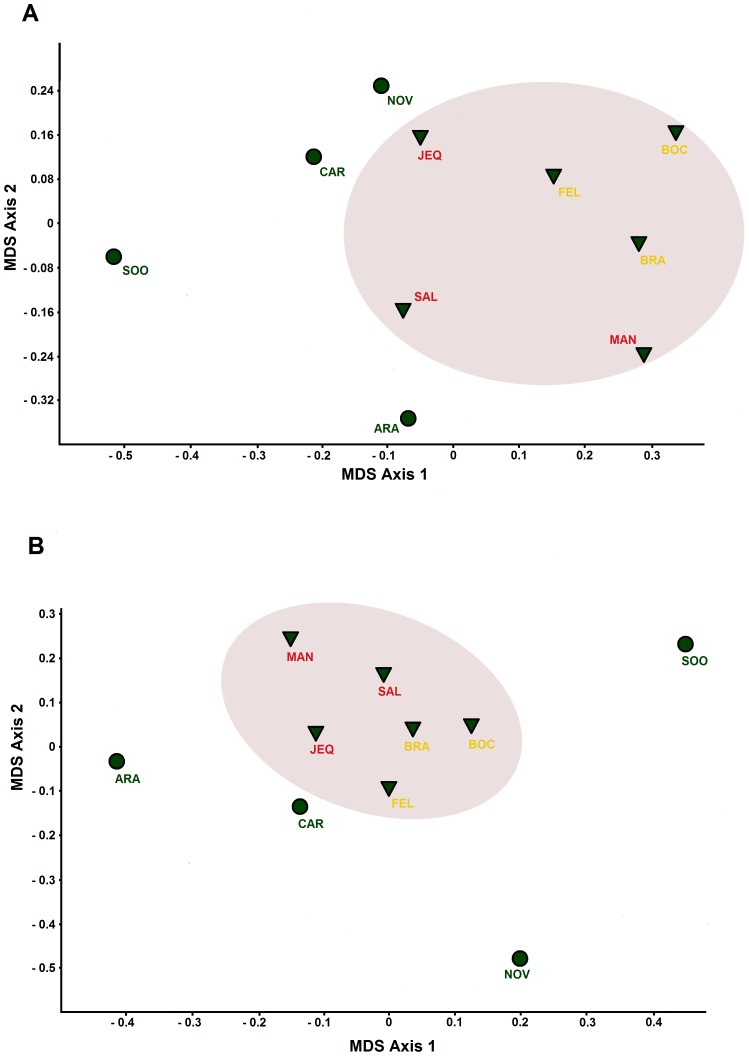
Scatterplots showing placement within non-dimensional metric scaling (NMDS) ordination space of bird species and parasite lineages compositions. NMDS is a nonparametric ordination technique that maps ranked data non-linearly onto ordination space using both taxa composition and abundance. The assemblage data (composition and relative abundance of taxa) were used to assign a position in ordination space to each sample. Samples with similar assemblages were positioned close to one another in ordination space, while samples with dissimilar assemblages were positioned further apart. NMDS ordination spaces of bird species (A) and parasite lineages (B) according to the habitat are represented. Symbols represent the two significant habitat grouping (circles: Atlantic rainforest; triangles: Brazilian savannah+seasonally dry forest).

Using the NMDS composition analyses we found that the parasite lineage composition in the Brazilian savannah and tropical dry forest was similar, and these were different from the lineage composition of the Atlantic rainforest (Table 2, Fig. 3). The composition analyses for the samples of the bird communities revealed a similar pattern, where the composition of the bird species sampled in Atlantic rainforest sites was different from that of the Brazilian savannah and tropical dry forest, reflecting the greater likeness of these habitats with respect to seasonality and forest density compared to the Atlantic rainforest area.

**Table 2:** Results of NMDS and ANOSIM for each hypothesis of lineage/bird species grouping proposed, with significant differences in bold.

Hypothesis		Parasite lineages		Bird species	
		Stress	p	Stress	p
***Habitat*** *	AF vs. BS vs. DF	0.2192	0.045	0.1654	0.1064
	AF+BS vs. DF	0.2158	0.6219	0.1637	0.8115
	AF+DF vs. BS	0.2148	0.5975	0.1652	0.2283
	**AF vs. BS+DF**	**0.2186**	**0.005**	**0.1643**	**0.029**

* AF = Atlantic rainforest, BS = Brazilian savannah, DF = tropical dry forest.

**Figure 4:**
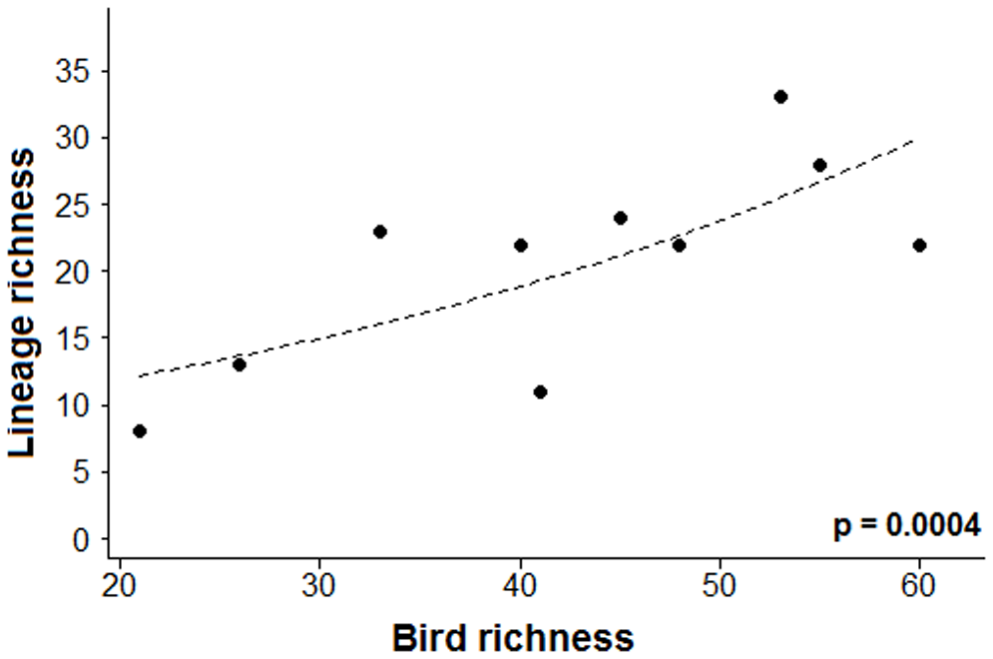
Relationship between bird richness and lineage richness. The significance of the correlation is shown in the bottom right of the figure.

Based on GLM analyses related to the lineage richness data, we could not find significant effects of the habitat type or habitat preference on lineage richness. However, we found that lineage richness among the sites was significantly associated with differences in bird richness of the local samplings (Table 3), and we verified that samples with more bird richness had greater lineage richness (Fig. 4). There was no significant association between prevalence and every explanatory variable tested was verified (p>0.05).

**Table 3:** Summary of the GLM results including deviance and residual information data.

Response variable	GLM Family^1^	Explanatory variables^2^	d.f.	Deviance difference	Res. d.f.	Residual deviance	*P*
***Richness***	Poisson	Null			9	28.833	
		Habitat	2	4.2040	7	24.629	0.1222
		**Bird Richness**	**1**	**12.420**	**6**	**12.209**	**0.0004**
		N	1	0.3552	5	11.854	0.5512
***Prevalence***	Quasibinomial	Null			18	163.16	
		Habitat	2	3.8382	16	159.32	0.8435
		Bird Richness	1	44.789	6	79.899	0.1301

1 Each GLM Family corresponds to a different GML error adjustment;

2 Results of interaction among explanatory variables and redundant results among the GLM’s sets were omitted;

3 Bird species able to colonize urban areas;

4 Bird species able to colonize open areas.

## Discussion

### Diversity and Distribution of Parasite Lineages

One of the first steps for understanding the evolution and dynamics of host-parasite communities is to characterize their diversity and structure [11]. In the present study, we performed the first molecular characterization of the malarial parasites in bird communities from southeast Brazil. The overall prevalence (35.3%) observed was similar to other community-level studies that used PCR screening approaches and were performed in tropical habitats of South America [16]–[17], [26]. However, the observed prevalence was higher than that found in previous studies that adopted only blood smear screening approaches [27]–[31]. Furthermore, considerable differences were observed between our prevalence data and the results reported by Sebaio et al. [30], which also sampled birds from southeast Brazil. We attribute these differences to the intrinsic efficacy variation of the screening methods, which has been observed in other studies [16], [32]–[33].

In addition, we found that *Plasmodium* prevalence was higher than *Haemoproteus* prevalence. Although this finding complies with the previous study performed in southeast Brazil, most of the studies in bird communities of South and North America show a higher prevalence of *Haemoproteus* [11], [17], [31], [34]–[36]. This contrary scenario presented by the malarial parasite community from southeast Brazil could be explained by differences of vector populations, Culicidae mosquitoes for *Plasmodium* and biting midges (*Culicoides*) and louse-flies (*Psedolynchia*, *Microlynchia* and *Ornithomyia*) for *Haemoproteus* [2], because the distribution of malaria vectors can be affected by vegetation type, rainfall patterns, mean temperatures, elevation, and geomorphology [37].

Sequencing the mtDNA cyt-*b* marker revealed a greater number of lineages compared to other regional surveys conducted in South America, Europe and Africa [26], [36], [38]–[40]; however, the population had similar diversity compared to those from Australo-Papuan region and India [10], [41]. The high lineage richness discovered in this study could be associated with the diverse population of host species in southeast Brazil because parasites were found in 132 different bird species. These findings suggest that the high diversity of hosts, in conjunction with the heterogeneity of environments, is a source of niches in which malarial parasites can specialize and diverge as distinct evolutionary lineages.

The variable host range observed for both *Plasmodium* and *Haemoproteus* species suggests that their lineages have different strategies of host exploration, ranging from complete generalism to high levels of host specificity. Alternatively, considering that each vector species has different patterns of distribution and behavior [37], [42], lineages that are limited to some vector species are restricted only to bird species that this vector could bite. Moreover, as the number of samples from each bird species was unbalanced, the results of the host range may overestimate cases of host specificity. In the present study, we verified that the *Plasmodium* lineages presented a broader spectrum of host species than did the *Haemoproteus* lineages. The evidence that indicated the restriction of *Haemoproteus* lineages at host level was also observed in community-level studies in Africa and Europe, suggesting that this could be an inherent attribute of the *Haemoproteus* genus [39], [43]–[44].

### Phylogenetic Relationships

The Bayesian phylogenetic inferences of the *Plasmodium* lineages revealed five major clades without a noticeable association with landscape or avian host family. However, associations among some subclades and particular host families and habitat types could be observed. First, the lineages that compose the most diverse subclade in Clade 1 almost exclusively infect birds from the Atlantic rainforest communities, despite these same bird species dwelling in other habitat types. This pattern suggests that some components of the Atlantic rainforest, such as suitable vectors, could limit lineages from this subclade to this particular habitat. Second, with the exception of the lineage GARUF01, all lineages from the subclade that includes the widespread *P. elongatum* were found in *Turdus* species as a host. The pairwise distances between the *P. elongatum* lineage GRW06 and all other lineages from this subclade ranged from 0.02% to 2.9%, suggesting that these lineages represent different lineages of *P. elongatum*. These lineages were established in the community after obtaining suitable conditions in Brazilian *Turdus* as a host species. Third, the majority of Clade 4 lineages were found in multiple bird species, suggesting that the generalist strategy of host exploration should be an inherited component of Clade 4 members instead of being an independently acquired characteristic for each species.

The traditional taxonomy of *Heamoproteus*, which is based on morphological characters observable by light microscopy, places the *Haemoproteus* species that infect Columbiformes in the sub-genus *Haemoproteus*, whereas all other *Haemoproteus* species should belong to the sub-genus *Parahaemoproteus* [2]. Recently, molecular evidence has supported the hypothesis that *Haemoproteus* (*Haemoproteus*) and *Haemoproteus* (*Parahaemoproteus*) can be considered distinct clades, maintaining the proposal of a sub-genus exclusively composed of species that infect Columbiformes [34], [45]. Our phylogenetic tree of *Haemoproteus* lineages also revealed the occurrence of two distinct clades corresponding to the Haemoproteus and Parahaemoproteus clades (Fig. 2). However, the lineage COTAL01, which belongs to the Haemoproteus clade, was found to infect two individuals of the *Coccyzus melacoryphus* (Cuculidae) species, a non-Columbiformes bird species. These findings introduce the possibility of the use of non-Columbiformes hosts by members of the sub-genus *Haemoproteus* (*Haemoproteus*), although a clear confirmation should include the morphological characterization of these parasites.

We observed that the pairwise genetic distances within the clades varied significantly, and in some cases, the intra-clade variation exceeded the variation among the clades. This pattern of variation hinders the establishment of divergence thresholds between lineage groups and prevents the proposition of putative *Plasmodium* and *Haemoproteus* species based on genetic distance values using the cyt-*b* marker.

### Associations among Habitat, Composition and Richness of Parasite Communities

The composition analyses of parasite communities revealed the same pattern presented by the bird sampling: a significant difference between birds of the Atlantic rainforest versus sampled species of birds from the Brazilian savannah and seasonally dry forest communities (Table 2 and Fig. 3). Atlantic rainforests are characterized by a high and constant rainfall and a narrow temperature variation throughout the year due to the influence of the coastal environment. Meanwhile, the Brazilian savannah and seasonally dry forests undergo more variation across the year from the two seasons, wet and dry [46]. Therefore, it is reasonable to hypothesize that the abiotic attributes of Atlantic rainforests may be associated with the differences in the composition of bird communities. Considering that our bird sampling represents a real picture of the composition of the bird communities, the presence of the same pattern for both bird and parasite communities indicates that the composition of a particular bird community affects the composition of malaria parasite lineages, most likely due to events of host specialization that occur over co-evolutionary interactions in a community.

The GLM analyses revealed that areas with a greater diversity of bird species also had high parasite richness, supporting the hypothesis that areas with high bird richness also have high parasite richness by providing more diverse host environments for different lineages of parasites to colonize. However, our sample size per site is positively correlated with lineage richness; thus, the uneven sampling effort among the sites may have influenced the results.

### Concluding Remarks

It is noteworthy that most of the lineages were reported here for the first time, indicating the singularity of the parasite community of the sampled areas and revealing the need for studies that address the relationship between these parasites and their hosts. Indeed, only 14 of the 110 lineages identified in this study had been described previously. The identification of previously described lineages is relevant for a better understanding of their spatial distribution and host range; for example, the *P. elongatum* GRW06 lineage that had already been found in birds from all other continents is described for the first time in South America, infecting three bird species. Moreover, further studies using greater and uniform sampling areas with well-founded evidence of differences in bird richness would be valuable to investigate the effects of bird community structure on the diversity of avian malaria communities and corroborate the results from this study.

## Methods

### Sampling Design

**Figure 5:**
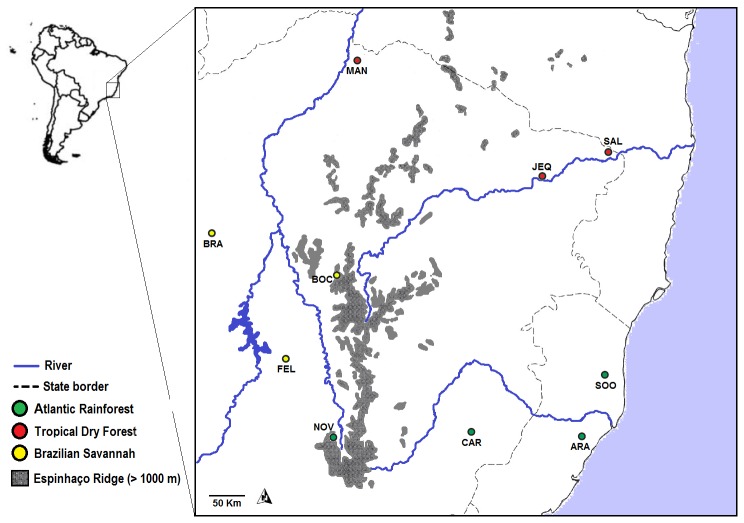
Map of southeast Brazil depicting the sampling sites and the delimitation of the highest areas of Espinhaço range. The names of the sampling sites are coded (ARA = Aracruz; BRA = Brasilândia; BOC = Bocaiúva; CAR = Caratinga; FEL = Felixlândia; JEQ = Jequitinhonha; MAN = Manga; NOV = Nova Lima; SAL = Salto da Divisa).

A total of 1,545 DNA samples from birds collected at 10 sites across the southeast Brazilian region were selected for this study (Table 1, Fig. 5). These DNA samples are part of the two bird DNA banks: LBEM DNA bank (maintained by FRS) and Malaria Lab Bird DNA bank (maintained by EMB). Samples from LBEM DNA bank were collected between 2000 and 2006 including samples from ARA, BOC, BRA, CAR, FEL, JEQ, NOV, SAL and SOO sites. Samples from Malaria Lab Bird DNA bank were obtained during 2010, including samples from MAN site. These sites were selected due to their representative bird communities with a variable richness of species comprising three different types of tropical ecosystems: Atlantic rainforest, Brazilian savannah and tropical dry forest [22]. The blood obtained from all birds, which were caught using mist nets, was stored in absolute alcohol in an ultra-freezer at −70C°. DNA extraction was performed with a phenol–chloroform–isoamilic alcohol protocol described in Sambrook et al. [47]. Samples from the LBEM DNA bank were collected between 2000 and 2006, whereas samples from the Malaria Lab Bird DNA bank were obtained during 2010.

The study was approved by the Ethics Committee in Animal Experimentation (CETEA), Federal University of Minas Gerais, Brazil (Protocol #254/2011).

### Molecular Analyses

DNA samples were initially screened for the presence of *Plasmodium* and/or *Haemoproteus* infections by PCR using primers 343F (5′GCTCACGCATCGCTTCT3′) and 496R (5′GACCGG-TCATTTTCTTTG3′) according to the protocol described by Fallon et al. [48]. The presence of a 195-bp band in acrylamide gels indicates the presence of *Plasmodium* and/or *Haemoproteus* infection. Subsequent to parasite screening, a 524 bp fragment of the mtDNA cytochrome b gene from the infected individuals was amplified by a nested-PCR using primers HaemNFI (CATATATTAAGAGAAITATGGAG) and HaemNR3 (ATAGAAAGATAAGAAATACCATTC) in a first amplification; and HaemF (ATGGTGCTTTCGATATATGCATG) and HaemR2 (GCATTATCTGGATGTGATAATGGT) in a second amplification, following protocols described by Hellgren et al. [49]. The PCR products were purified using a solution of 20% polyethylene-glycol 8000 and 2.5 M NaCl according to the methods of Sambrook et al. [47]. After purification, the PCR products were sequenced in both directions using the BigDye Terminator Kit v3 (Applied Biosystems, Foster City, CA, USA) using an ABI3100® automated sequencer (Applied Biosystems, Foster City, CA, USA).

The sequences were assembled and checked for quality using Phred v.0.20425 [50]–[51] and Phrap v.0.990319 [52]. The assembled chromatograms were carefully checked and edited using Consed 23.0 [53]. Sequences showing double peaks were examined for multiple infections by cloning (TOPO-cloning kit, Invitrogen®) and sequencing. We sequenced six clones from each sample for which we observed a likely co-infection. Sequences were aligned using the Clustal W algorithm implemented in MEGA version 5 [54]. As there is no consensus on how to delimit haemosporidian species based on cyt-b sequences [55], we considered each haplotype as an independent lineage and our unit of richness for further analyses. The genus of each lineage was inferred by the closest sequence matches in Genbank using an NCBI nucleotide Blast search. In an attempt to assign the sequences to describe parasite lineages, we compared the sequences with records in the Genbank and MalAvi databases [56], which contain cyt-b data for most of published avian haemosporidian parasite lineages. Observed lineages that were not present in the MalAvi database were considered new lineages. All sequences were deposited in Genbank (see Table S1).

### Phylogenetic and Statistical Analyses

Bayesian analyses were implemented to infer the phylogenetic relationships among cyt-b lineages. MrBayes version 3.1.2 [57] was used to run two Markov chains simultaneously for 3 million generations that were sampled every 100 generations. The first 7500 trees (25%) were discarded as a “burn-in” step and the remaining trees were used to calculate the posterior probabilities. The *Plasmodium* and *Haemoproteus* lineages were analyzed separately because there is no consensus about the monophyly of both genera [34], [58]. A sequence of *P. malariae* was used as an out-group based on recent evidence suggesting that the *Plasmodium* genus is polyphyletic, and *Plasmodium* species that infect mammals form a sister clade with the clade that harbors the groups studied here [58]. Sequence divergence within and among the major *Plasmodium* and *Haemoproteus* clades was calculated using uncorrected-P distance in MEGA version 5 [54].

For statistical analyses, we first used a non-metric multidimensional scaling (NMDS) model to search for overall differences in parasite lineage compositions between the following habitats: Atlantic rainforest, Brazilian savannah and tropical dry forest. We also used an analysis of similarities (ANOSIM) to test the significance of the differences found by the NMDS. The composition analyses were performed using the software PAST [59]. We performed similar composition analyses for the bird samples to identify the possible effects of bird sampling in the NMDS results for parasite lineages.

Next, we applied generalized linear models (GLMs) to identify possible effects of the type of habitat and bird richness (explanatory variables) on the prevalence of infection and lineage richness (response variables). All GLMs were submitted to residual analyses to evaluate the adequacy of the error distribution. Minimum adequate models were generated by a stepwise omission of non-significant terms. The GLMs were performed with the software R [60]. Additionally, for prevalence analyses, we excluded data from bird species with less than 5 observations to avoid sampling bias.

## Supporting Information

Figure S1 Observed host range of the *Plasmodium* and *Haemoproteus* lineages.
(TIF)

Table S1 Summary of lineage parasite information, including Genbank accession numbers.
(DOCX)
