# Supplementary figures and images for: Exploring the Diversity and Distribution of Neotropical Avian Malaria Parasites – A Molecular Survey from Southeast Brazil
Source: PLoS One. 2013 Mar 1;8(3):e57770. doi: 10.1371/journal.pone.0057770 (PMC3585926; doi:10.1371/journal.pone.0057770)

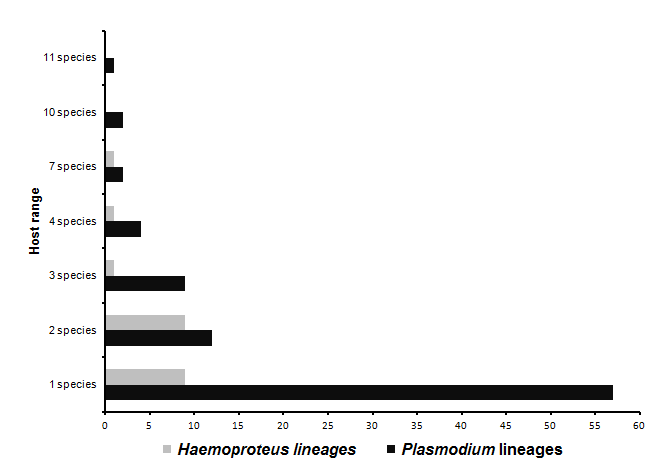

Supplement: Figure S1 — Observed host range of the Plasmodium and Haemoproteus lineages. (TIF) [file pone.0057770.s001.tif]
