# Supplementary material for: Exploring the Diversity and Distribution of Neotropical Avian Malaria Parasites – A Molecular Survey from Southeast Brazil
Source: PLoS One. 2013 Mar 1;8(3):e57770. doi: 10.1371/journal.pone.0057770 (PMC3585926; doi:10.1371/journal.pone.0057770)
Supplement: Table S1 — Summary of lineage parasite information, including Genbank accession numbers. (DOCX) [file pone.0057770.s002.docx]

Table S1. Summary of lineage parasite information, including Genbank accession numbers.

| Lineage | Parasite genus | Host Species*  Scientific name Common name | | | Host Family | | Site | | Genbank | |
| --- | --- | --- | --- | --- | --- | --- | --- | --- | --- | --- |
| ANGAL01 | *Plasmodium* | *Antilophia galeata* | Helmeted Manakin | | Pipridae | | B. de Minas | | JX029873 | |
| BAFLA01 | *Plasmodium* | *Conopophaga lineata* | Rufous Gnateater | | Conopophagidae | | Jequitinhonha | | JX021453 | |
|  |  | *Basileuterus flaveolus* | Flavescent Warbler | | Parulidae | | Nova Lima | |  |  |
|  |  | *Basileuterus flaveolus* (2) | Flavescent Warbler | | Parulidae | | Jequitinhonha | |  |  |
| BAFLA02 | *Haemoproteus* | *Tyrannus melancholicus* | Tropical Kingbird | | Tyrannidae | | Salto da Divisa | | JX029903 | |
|  |  | *Basileuterus flaveolus* | Flavescent Warbler | | Parulidae | | Felixlândia | |  |  |
| BAFLA03 | *Plasmodium* | *Basileuterus culicivorus* (2) | Golden-crowned Warbler | | Parulidae | | Bocaiúva | | JX021476 | |
|  |  | *Basileuterus flaveolus* (3) | Flavescent Warbler | | Parulidae | | Jequitinhonha | |  |  |
|  |  | *Basileuterus flaveolus* | Flavescent Warbler | | Parulidae | | Bocaiúva | |  |  |
|  |  | *Celeus flavescens* | Blond-crested Woodpecker | | Picidae | | B. de Minas | |  |  |
|  |  | *Dryocopus lineatus* | Lineated Woodpecker | | Picidae | | B. de Minas | |  |  |
|  |  | *Euphonia violacea* | Violaceous Euphonia | | Fringillidae | | Salto da Divisa | |  |  |
|  |  | *Hylophilus amaurocephalus* | Gray-eyed Greenlet | | Vireonidae | | Caratinga | |  |  |
|  |  | *Hylophilus amaurocephalus* | Gray-eyed Greenlet | | Vireonidae | | Manga | |  |  |
|  |  | *Leptopogon amaurocephalus* (2) | Sepia-capped Flycatcher | | Tyrannidae | | Caratinga | |  |  |
|  |  | *Myiarchus tyrannulus* | Brown-crested Flycatcher | | Tyrannidae | | Manga | |  |  |
|  |  | *Trichothraupis melanops* (2) | Black-goggled Tanager | | Thraupidae | | Caratinga | |  |  |
|  |  | *Turdus rufiventris* | Rufous-bellied Thrush | | Turdidae | | Caratinga | |  |  |
| BAFLA04 | *Plasmodium* | *Tiaris fuliginosus* | Sooty Grassquit | | Emberizidae | | Salto da Divisa | | JX029861 | |
|  |  | *Paroaria dominicana* | Red-cowled Cardinal | | Emberizidae | | Salto da Divisa | |  |  |
|  |  | *Basileuterus flaveolus* (3) | Flavescent Warbler | | Parulidae | | Felixlândia | |  |  |
| BAFLA05 | *Plasmodium* | *Basileuterus flaveolus* | Flavescent Warbler | | Parulidae | | B. de Minas | | JX029890 | |
| BAHYP01 | *Plasmodium* | *Basileuterus hypoleucus* (3) | White-bellied Warbler | | Parulidae | | B. de Minas | | JX029875 | |
|  |  | *Coereba flaveola* | Bananaquit | | Coerebidae | | Felixlândia | |  |  |
|  |  | *Coryphospingus pileatus* | Grey Pileated Finch | | Emberizidae | | Felixlândia | |  |  |
| CAOBS01 | *Plasmodium* | *Camptostoma obsoletum* | Southern Beardless-Tyrannulet | | Tyrannidae | | Felixlândia | | JX029879 | |
|  |  | *Camptostoma obsoletum* (2) | Southern Beardless-Tyrannulet | | Tyrannidae | | B. de Minas | |  |  |
|  |  | *Gnorimopsar chopi* | Chopi Blackbird | | Icteridae | | B. de Minas | |  |  |
|  |  | *Thamnophilus caerulescens* | Variable Antshrike | | Thamnophilidae | | Nova Lima | |  |  |
| CARUF01 | *Haemoproteus* | *Casiornis rufus* (5) | Rufous Casiornis | | Tyrannidae | | Felixlândia | | JX029902 | |
|  |  | *Coryphospingus pileatus* | Grey Pileated Finch | | Emberizidae | | Salto da Divisa | |  |  |
| CHCAM01 | *Plasmodium* | *Chamaeza campanisona* | Short-tailed Antthrush | | Formicariidae | | Jequitinhonha | | JX021464 | |
| COLIN01 | *Plasmodium* | *Conopophaga lineata* | Rufous Gnateater | | Conopophagidae | | Caratinga | | JX021479 | |
|  |  | *Conopophaga lineata* (5) | Rufous Gnateater | | Conopophagidae | | Nova Lima | |  |  |
| COLIN02 | *Plasmodium* | *Conopophaga lineata* | Rufous Gnateater | | Conopophagidae | | Caratinga | | JX021480 | |
| COLIN03 | *Plasmodium* | *Conopophaga lineata* | Rufous Gnateater | | Conopophagidae | | Caratinga | | JX021481 | |
| COLIN04 | *Plasmodium* | *Conopophaga lineata* | Rufous Gnateater | | Conopophagidae | | Caratinga | | JX021482 | |
| COLIN05 | *Plasmodium* | *Conopophaga lineata* (5) | Rufous Gnateater | | Conopophagidae | | Caratinga | | JX021489 | |
|  |  | *Conopophaga lineata* (5) | Rufous Gnateater | | Conopophagidae | | Nova Lima | |  |  |
|  |  | *Leptopogon amaurocephalus* (2) | Sepia-capped Flycatcher | | Tyrannidae | | Nova Lima | |  |  |
|  |  | *Philydor rufum* (2) | Buff-fronted Foliage-gleaner | | Furnariidae | | Nova Lima | |  |  |
|  |  | *Polioptila plumbea* (3) | Tropical Gnatcatcher | | Polioptilidae | | Manga | |  |  |
|  |  | *Thraupis sayaca* | Sayaca Tanager | | Thraupidae | | Aracruz | |  |  |
|  |  | *Tolmomyias flaviventris* | Yellow-breasted Flycatcher | | Tyrannidae | | Jequitinhonha | |  |  |
|  |  | *Trichothraupis melanops* | Black-goggled Tanager | | Thraupidae | | Caratinga | |  |  |
| COLIN06 | *Plasmodium* | *Conopophaga lineata* | Rufous Gnateater | | Conopophagidae | | Nova Lima | | JX021490 | |
| COLIN07 | *Plasmodium* | *Conopophaga lineata* (2) | Rufous Gnateater | | Conopophagidae | | Nova Lima | | JX021491 | |
| COLIN08 | *Plasmodium* | *Conopophaga lineata* | Rufous Gnateater | | Conopophagidae | | Nova Lima | | JX021492 | |
| COLIN09 | *Plasmodium* | *Conopophaga lineata* | Rufous Gnateater | | Conopophagidae | | Nova Lima | | JX021493 | |
| COLIN10 | *Plasmodium* | *Conopophaga lineata* | Rufous Gnateater | | Conopophagidae | | Nova Lima | | JX021494 | |
| COLIN11 | *Plasmodium* | *Conopophaga lineata* (5) | Rufous Gnateater | | Conopophagidae | | Nova Lima | | JX029899 | |
| COLIN12 | *Plasmodium* | *Conopophaga lineata* | Rufous Gnateater | | Conopophagidae | | Nova Lima | | JX021495 | |
| COLIN13 | *Plasmodium* | *Conopophaga lineata* | Rufous Gnateater | | Conopophagidae | | Nova Lima | | JX021496 | |
| COLIN14 | *Plasmodium* | *Conopophaga lineata* | Rufous Gnateater | | Conopophagidae | | Nova Lima | | JX021497 | |
| COLIN15 | *Plasmodium* | *Conopophaga lineata* | Rufous Gnateater | | Conopophagidae | | Nova Lima | | JX021451 | |
| COLIN16 | *Plasmodium* | *Basileuterus flaveolus* | Flavescent Warbler | | Parulidae | | Jequitinhonha | | JX021452 | |
|  |  | *Conopophaga lineata* | Rufous Gnateater | | Conopophagidae | | Nova Lima | |  |  |
|  |  | *Conopophaga lineata* (2) | Rufous Gnateater | | Conopophagidae | | Jequitinhonha | |  |  |
| COMEL01 | *Plasmodium* | *Conopophaga melanops* | Black-cheeked Gnateater | | Conopophagidae | | Caratinga | | JX021455 | |
|  |  | *Conopophaga melanops* | Black-cheeked Gnateater | | Conopophagidae | | Salto da Divisa | |  |  |
| COPIL01 | *Plasmodium* | *Coryphospingus pileatus* (3) | Grey Pileated Finch | | Emberizidae | | Bocaiúva | | JX021471 | |
|  |  | *Nonnula rubecula* | Rusty-breasted Nunlet | | Bucconidae | | B. de Minas | |  |  |
|  |  | *Troglodytes musculus* (2) | Southern House Wren | | Troglodytidae | | Salto da Divisa | |  |  |
|  |  | *Volatinia jacarina* (2) | Blue-black Grassquit | | Emberizidae | | Salto da Divisa | |  |  |
| COPIL02 | *Plasmodium* | *Coryphospingus pileatus* | Grey Pileated Finch | | Emberizidae | | Jequitinhonha | | JX029863 | |
| COSQU01 | *Haemoproteus* | *Claravis pretiosa* | Blue Ground-Dove | | Columbidae | | Bocaiúva | | JX029921 | |
|  |  | *Claravis pretiosa* | Blue Ground-Dove | | Columbidae | | B. de Minas | |  |  |
|  |  | *Columbina squammata* (2) | Scaled Dove | | Columbidae | | Felixlândia | |  |  |
|  |  | *Columbina squammata* (2) | Scaled Dove | | Columbidae | | Manga | |  |  |
| COTAL01 | *Haemoproteus* | *Coccyzus melacoryphus* (2) | Dark-billed Cuckoo | | Cuculidae | | Salto da Divisa | | JX029904 | |
|  |  | *Columbina talpacoti* | Ruddy Ground-Dove | | Columbidae | | Salto da Divisa | |  |  |
|  |  | *Columbina talpacoti* | Ruddy Ground-Dove | | Columbidae | | Manga | |  |  |
| DENPET03 | *Plasmodium* | *Basileuterus flaveolus* (3) | Flavescent Warbler | | Parulidae | | Felixlândia | | JX021474 | |
|  |  | *Basileuterus flaveolus* | Flavescent Warbler | | Parulidae | | B. de Minas | |  |  |
|  |  | *Cantorchilus longirostris* | Long-billed Wren | | Troglodytidae | | Manga | |  |  |
|  |  | *Coereba flaveola* (2) | Bananaquit | | Coerebidae | | Felixlândia | |  |  |
|  |  | *Coereba flaveola* | Bananaquit | | Coerebidae | | B. de Minas | |  |  |
|  |  | *Formicivora melanogaster* | Black-bellied Antwren | | Thamnophilidae | | Bocaiúva | |  |  |
|  |  | *Pachyramphus polychopterus* | White-winged Becard | | Tyrannidae | | Felixlândia | |  |  |
|  |  | *Parula pitiayumi* | Tropical Parula | | Parulidae | | B. de Minas | |  |  |
|  |  | *Trichothraupis melanops* | Black-goggled Tanager | | Thraupidae | | Caratinga | |  |  |
|  |  | *Turdus leucomelas* | Pale-breasted Thrush | | Turdidae | | B. de Minas | |  |  |
|  |  | *Turdus rufiventris* | Rufous-bellied Thrush | | Turdidae | | Salto da Divisa | |  |  |
|  |  | *Zonotrichia capensis* | Rufous-collared Sparrow | | Emberizidae | | Aracruz | |  |  |
| DETUR01 | *Plasmodium* | *Dendrocincla turdina* (3) | Plain-winged Woodcreeper | | Dendrocolaptidae | | Sooretama | | JX029892 | |
| ELALB01 | *Haemoproteus* | *Capsiempis flaveola* (2) | Yellow Tyrannulet | | Tyrannidae | | Salto da Divisa | | JX029900 | |
|  |  | *Casiornis fuscus* (3) | Ash-throated Casiornis | | Tyrannidae | | Jequitinhonha | |  |  |
|  |  | *Casiornis fuscus* | Ash-throated Casiornis | | Tyrannidae | | Salto da Divisa | |  |  |
|  |  | *Dendrocolaptes platyrostris* | Planalto Woodcreeper | | Dendrocolaptidae | | B. de Minas | |  |  |
|  |  | *Myiarchus tuberculifer* | Dusky-capped Flycatcher | | Tyrannidae | | Sooretama | |  |  |
|  |  | *Myiodynastes maculatus* (2) | Streaked Flycatcher | | Tyrannidae | | Salto da Divisa | |  |  |
|  |  | *Pitangus sulphuratus* | Great Kiskadee | | Tyrannidae | | Salto da Divisa | |  |  |
|  |  | *Tolmomyias flaviventris* (3) | Yellow-breasted Flycatcher | | Tyrannidae | | Salto da Divisa | |  |  |
| ELFLA01 | *Haemoproteus* | *Elaenia flavogaster* | Yellow-bellied Elaenia | | Tyrannidae | | Aracruz | | JX029917 | |
|  |  | *Tyrannus melancholicus* | Tropical Kingbird | | Tyrannidae | | Aracruz | |  |  |
| EUCHL01 | *Plasmodium* | *Euphonia chlorotica* | Purple-throated Euphonia | | Fringillidae | | Bocaiúva | | JX029882 | |
| EUVIO01 | *Plasmodium* | *Euphonia violacea* | Violaceous Euphonia | | Fringillidae | | Salto da Divisa | | JX021469 | |
| FOCOL01 | *Plasmodium* | *Formicarius colma* (2) | Rufous-capped Antthrush | | Formicariidae | | Sooretama | | JX029894 | |
| FOCOL02 | *Plasmodium* | *Formicarius colma* | Rufous-capped Antthrush | | Formicariidae | | Sooretama | | JX029895 | |
| FOMEL01 | *Plasmodium* | *Formicivora melanogaster* | Black-bellied Antwren | | Thamnophilidae | | Bocaiúva | | JX029870 | |
| FOSER01 | *Plasmodium* | *Drymophila squamata* | Scaled Antbird | | Thamnophilidae | | Caratinga | | JX021465 | |
|  |  | *Formicivora melanogaster* | Black-bellied Antwren | | Thamnophilidae | | Bocaiúva | |  |  |
|  |  | *Formicivora serrana* | Serra Antwren | | Thamnophilidae | | Caratinga | |  |  |
| FULEU01 | *Plasmodium* | *Furnarius leucopus* | Pale-legged Hornero | | Furnariidae | | Salto da Divisa | | JX029901 | |
|  |  | *Furnarius leucopus* | Pale-legged Hornero | | Furnariidae | | Manga | |  |  |
|  |  | *Pachyramphus polychopterus* | White-winged Becard | | Tyrannidae | | Salto da Divisa | |  |  |
| FURUF01 | *Plasmodium* | *Furnarius rufus* | Rufous Hornero | | Furnariidae | | B. de Minas | | JX029887 | |
| GARUF01 | *Plasmodium* | *Galbula ruficauda* | Rufous-tailed Jacamar | | Galbulidae | | B. de Minas | | JX029880 | |
| GRW06 | *Plasmodium* | *Turdus leucomelas* | Pale-breasted Thrush | | Turdidae | | Jequitinhonha | | JX029877 | |
|  |  | *Basileuterus hypoleucus* | White-bellied Warbler | | Parulidae | | B. de Minas | |  |  |
|  |  | *Malacoptila striata* | Crescent-chested Puffbird | | Bucconidae | | Aracruz | |  |  |
|  |  | *Turdus leucomelas* | Pale-breasted Thrush | | Turdidae | | Aracruz | |  |  |
| HYREC01 | *Plasmodium* | *Hylocryptus rectirostris* | Chestnut-capped Foliage-gleaner | | Furnariidae | | B. de Minas | | JX029866 | |
| ICTCAY01 | *Plasmodium* | *Basileuterus hypoleucus* (2) | White-bellied Warbler | | Parulidae | | Nova Lima | | JX021483 | |
| ILMIL01 | *Plasmodium* | *Ilicura militaris* (2) | Pin-tailed Manakin | | Pipridae | | Nova Lima | | JX021478 | |
|  |  | *Thamnophilus caerulescens* | Variable Antshrike | | Thamnophilidae | | Nova Lima | |  |  |
| LEAMA01 | *Plasmodium* | *Conopophaga lineata* (2) | Rufous Gnateater | | Conopophagidae | | Jequitinhonha | | JX021454 | |
|  |  | *Leptopogon amaurocephalus* (6) | Sepia-capped Flycatcher | | Tyrannidae | | Caratinga | |  |  |
|  |  | *Leptopogon amaurocephalus* (3) | Sepia-capped Flycatcher | | Tyrannidae | | Felixlândia | |  |  |
| LERUF01 | *Haemoproteus* | *Leptotila rufaxilla* | Gray-fronted Dove | | Columbidae | | Jequitinhonha | | JX029909 | |
|  |  | *Leptotila verreauxi* | White-tipped Dove | | Columbidae | | Manga | |  |  |
| MASTR01 | *Plasmodium* | *Malacoptila striata* | Crescent-chested Puffbird | | Bucconidae | | Jequitinhonha | | JX029876 | |
| MOBON01 | *Plasmodium* | *Molothrus bonariensis* | Shiny Cowbird | | Icteridae | | B. de Minas | | JX029889 | |
| MYCAN01 | *Plasmodium* | *Myiopagis caniceps* | Gray Elaenia | | Tyrannidae | | Caratinga | | JX029883 | |
| MYITYR01 | *Plasmodium* | *Myiarchus tuberculifer* (2) | Dusky-capped Flycatcher | | Tyrannidae | | Salto da Divisa | | JX021461 | |
|  |  | *Myiarchus tyrannulus* (4) | Brown-crested Flycatcher | | Tyrannidae | | Salto da Divisa | |  |  |
|  |  | *Myiodynastes maculatus* (3) | Streaked Flycatcher | | Tyrannidae | | B. de Minas | |  |  |
|  |  | *Myiodynastes maculatus* (2) | Streaked Flycatcher | | Tyrannidae | | Felixlândia | |  |  |
| MYMAC01 | *Haemoproteus* | *Myiodynastes maculatus* | Streaked Flycatcher | | Tyrannidae | | B. de Minas | | JX029914 | |
| MYSWA01 | *Plasmodium* | *Myiarchus ferox* | Short-crested Flycatcher | | Tyrannidae | | B. de Minas | | JX029885 | |
|  |  | *Myiarchus swainsoni* (2) | Swainson's Flycatcher | | Tyrannidae | | Bocaiúva | |  |  |
|  |  | *Myiobius barbatus* | Whiskered Flycatcher | | Tyrannidae | | Bocaiúva | |  |  |
| NEPIL01 | *Haemoproteus* | *Nemosia pileata* | Hooded Tanager | | Thraupidae | | Salto da Divisa | | JX029906 | |
|  |  | *Pheugopedius genibarbis* | Moustached Wren | | Troglodytidae | | Salto da Divisa | |  |  |
| PACPEC02 | *Haemoproteus* | *Hemitriccus margaritaceiventer* (5) | | Pearly-vented Tody-tyrant | | Tyrannidae | Felixlândia | JX029911 | |  |
|  |  | *Nemosia pileata* | Hooded Tanager | | Thraupidae | | Felixlândia | |  | |
| PADOM09 | *Plasmodium* | *Basileuterus culicivorus* | Golden-crowned Warbler | | Parulidae | | Bocaiúva | | JX025076 | |
|  |  | *Cnemotriccus fuscatus* (3) | Fuscous Flycatcher | | Tyrannidae | | Jequitinhonha | |  |  |
|  |  | *Cnemotriccus fuscatus* (3) | Fuscous Flycatcher | | Tyrannidae | | Felixlândia | |  |  |
|  |  | *Cnemotriccus fuscatus* | Fuscous Flycatcher | | Tyrannidae | | B. de Minas | |  |  |
|  |  | *Elaenia cristata* (2) | Plain-crested Elaenia | | Tyrannidae | | Felixlândia | |  |  |
|  |  | *Lathrotriccus euleri* (2) | Euler's Flycatcher | | Tyrannidae | | Felixlândia | |  |  |
|  |  | *Lathrotriccus euleri* (2) | Euler's Flycatcher | | Tyrannidae | | Bocaiúva | |  |  |
|  |  | *Myiopagis viridicata* | Greenish Elaenia | | Tyrannidae | | Jequitinhonha | |  |  |
|  |  | *Myiopagis viridicata* (2) | Euler's Flycatcher | | Tyrannidae | | B. de Minas | |  |  |
|  |  | *Myiophobus fasciatus* | Bran-colored Flycatcher | | Tyrannidae | | Salto da Divisa | |  |  |
|  |  | *Pheugopedius genibarbis* | Moustached Wren | | Troglodytidae | | Salto da Divisa | |  |  |
|  |  | *Pitangus sulphuratus* | Great Kiskadee | | Tyrannidae | | Salto da Divisa | |  |  |
|  |  | *Trichothraupis melanops* | Black-goggled Tanager | | Thraupidae | | Caratinga | |  |  |
|  |  | *Troglodytes musculus* | Southern House Wren | | Troglodytidae | | Aracruz | |  |  |
|  |  | *Tyrannus melancholicus* | Tropical Kingbird | | Tyrannidae | | Aracruz | |  |  |
| PADOM11 | *Plasmodium* | *Basileuterus flaveolus* | Flavescent Warbler | | Parulidae | | Felixlândia | | JX021463 | |
|  |  | *Coryphospingus pileatus* | Grey Pileated Finch | | Emberizidae | | Felixlândia | |  |  |
|  |  | *Dacnis cayana* | Blue Dacnis | | Thraupidae | | B. de Minas | |  |  |
|  |  | *Polioptila plumbea* | Tropical Gnatcatcher | | Polioptilidae | | Bocaiúva | |  |  |
|  |  | *Saltator similis* (2) | Green-winged Saltator | | Cardinalidae | | Nova Lima | |  |  |
|  |  | *Sittasomus griseicapillus* | Olivaceous Woodcreeper | | Dendrocolaptidae | | B. de Minas | |  |  |
|  |  | *Trichothraupis melanops* (3) | Black-goggled Tanager | | Thraupidae | | Jequitinhonha | |  |  |
| PADOM17 | *Plasmodium* | *Tiaris fuliginosus* | Sooty Grassquit | | Emberizidae | | Salto da Divisa | | JX021470 | |
| PAPOL01 | *Haemoproteus* | *Nemosia pileata* | Hooded Tanager | | Thraupidae | | Salto da Divisa | | JX029905 | |
|  |  | *Nyctidromus albicollis* | Common Pauraque | | Caprimulgidae | | Salto da Divisa | |  |  |
|  |  | *Pachyramphus polychopterus* | White-winged Becard | | Tyrannidae | | Salto da Divisa | |  |  |
|  |  | *Paroaria dominicana* | Red-cowled Cardinal | | Emberizidae | | Salto da Divisa | |  |  |
| PAPOL02 | *Haemoproteus* | *Pachyramphus polychopterus* | White-winged Becard | | Tyrannidae | | Salto da Divisa | | JX029907 | |
| PAPOL03 | *Haemoproteus* | *Pachyramphus polychopterus* | White-winged Becard | | Tyrannidae | | Salto da Divisa | | JX029912 | |
|  |  | *Pachyramphus polychopterus* (2) | White-winged Becard | | Tityridae | | Manga | |  |  |
| PAPOL04 | *Haemoproteus* | *Pachyramphus polychopterus* | White-winged Becard | | Tyrannidae | | Salto da Divisa | | JX029913 | |
| PAPOL05 | *Plasmodium* | *Pachyramphus polychopterus* | White-winged Becard | | Tyrannidae | | Salto da Divisa | | JX029884 | |
| PAPOL06 | *Plasmodium* | *Pachyramphus polychopterus* | White-winged Becard | | Tyrannidae | | B. de Minas | | JX029888 | |
| PESA01 | *Plasmodium* | *Phaeomyias murina* | Mouse-colored Tyrannulet | | Tyrannidae | | Salto da Divisa | | JX029862 | |
| PHPAT01 | *Plasmodium* | *Ammodramus humeralis* | Grassland Sparrow | | Emberizidae | | Salto da Divisa | | JX025077 | |
|  |  | *Basileuterus flaveolus* | Flavescent Warbler | | Parulidae | | Felixlândia | |  |  |
|  |  | *Phaeomyias murina* | Mouse-colored Tyrannulet | | Tyrannidae | | Salto da Divisa | |  |  |
|  |  | *Phaeomyias murina* | Mouse-colored Tyrannulet | | Tyrannidae | | B. de Minas | |  |  |
| PISUL01 | *Haemoproteus* | *Dendrocolaptes platyrostris* | Planalto Woodcreeper | | Dendrocolaptidae | | B. de Minas | | JX029919 | |
|  |  | *Pitangus sulphuratus* | Great Kiskadee | | Tyrannidae | | Salto da Divisa | |  |  |
|  |  | *Thamnophilus ambiguus* | Sooretama Slaty-Antshrike | | Thamnophilidae | | Salto da Divisa | |  |  |
| PYLEU01 | *Plasmodium* | *Dysithamnus plumbeus* (7) | Plumbeous Antvireo | | Thamnophilidae | | Caratinga | | JX021484 | |
|  |  | *Pyriglena leucoptera* (2) | White-shouldered Fire-eye | | Thamnophilidae | | Caratinga | |  |  |
|  |  | *Pyriglena leucoptera* | White-shouldered Fire-eye | | Thamnophilidae | | Sooretama | |  |  |
|  |  | *Pyriglena leucoptera* (2) | White-shouldered Fire-eye | | Thamnophilidae | | Salto da Divisa | |  |  |
|  |  | *Pyriglena leucoptera* (2) | White-shouldered Fire-eye | | Thamnophilidae | | Bocaiúva | |  |  |
|  |  | *Pyriglena leucoptera* | White-shouldered Fire-eye | | Thamnophilidae | | Nova Lima | |  |  |
| PYLEU02 | *Plasmodium* | *Pyriglena leucoptera* | White-shouldered Fire-eye | | Thamnophilidae | | Nova Lima | | JX021485 | |
| PYLEU03 | *Plasmodium* | *Pyriglena leucoptera* | White-shouldered Fire-eye | | Thamnophilidae | | Nova Lima | | JX021486 | |
| PYLEU04 | *Plasmodium* | *Pyriglena leucoptera* | White-shouldered Fire-eye | | Thamnophilidae | | Salto da Divisa | | JX021487 | |
| PYLEU05 | *Plasmodium* | *Pyriglena leucoptera* | White-shouldered Fire-eye | | Thamnophilidae | | Salto da Divisa | | JX021488 | |
| SYCIN01 | *Haemoproteus* | *Synallaxis cinerea* | Bahia Spinetail | | Furnariidae | | Jequitinhonha | | JX029910 | |
| TARUF01 | *Plasmodium* | *Basileuterus flaveolus* | Flavescent Warbler | | Parulidae | | Felixlândia | | JX021475 | |
|  |  | *Saltator similis* | Green-winged Saltator | | Cardinalidae | | Felixlândia | |  |  |
|  |  | *Tachyphonus rufus* (2) | White-lined Tanager | | Thraupidae | | B. de Minas | |  |  |
|  |  | *Tangara cayana* | Burnished-buff Tanager | | Thraupidae | | Caratinga | |  |  |
| THAMB01 | *Plasmodium* | *Thamnophilus ambiguus* (6) | Sooretama Slaty-Antshrike | | Thamnophilidae | | Jequitinhonha | | JX021456 | |
| THAMB02 | *Plasmodium* | *Thamnophilus ambiguus* (5) | Sooretama Slaty-Antshrike | | Thamnophilidae | | Caratinga | | JX021458 | |
|  |  | *Thamnophilus ambiguus* (15) | Sooretama Slaty-Antshrike | | Thamnophilidae | | Salto da Divisa | |  |  |
|  |  | *Thamnophilus ambiguus* (2) | Sooretama Slaty-Antshrike | | Thamnophilidae | | Sooretama | |  |  |
| THAMB03 | *Plasmodium* | *Thamnophilus ambiguus* (4) | Sooretama Slaty-Antshrike | | Thamnophilidae | | Salto da Divisa | | JX021459 | |
| THAMB04 | *Plasmodium* | *Thamnophilus ambiguus* | Sooretama Slaty-Antshrike | | Thamnophilidae | | Bocaiúva | | JX029864 | |
| THAMB05 | *Plasmodium* | *Thamnophilus ambiguus* | Sooretama Slaty-Antshrike | | Thamnophilidae | | Bocaiúva | | JX029865 | |
| THAMB06 | *Plasmodium* | *Thamnophilus ambiguus* | Sooretama Slaty-Antshrike | | Thamnophilidae | | Bocaiúva | | JX029867 | |
| THAMB07 | *Plasmodium* | *Thamnophilus ambiguus* | Sooretama Slaty-Antshrike | | Thamnophilidae | | Bocaiúva | | JX029868 | |
| THAMB08 | *Plasmodium* | *Thamnophilus ambiguus* (2) | Sooretama Slaty-Antshrike | | Thamnophilidae | | Bocaiúva | | JX029869 | |
|  |  | *Thamnophilus ambiguus* | Sooretama Slaty-Antshrike | | Thamnophilidae | | Caratinga | |  |  |
|  |  | *Thamnophilus ambiguus* | Sooretama Slaty-Antshrike | | Thamnophilidae | | Sooretama | |  |  |
| THAMB09 | *Plasmodium* | *Pheugopedius genibarbis* | Moustached Wren | | Troglodytidae | | Aracruz | | JX029893 | |
|  |  | *Thamnophilus ambiguus* | Sooretama Slaty-Antshrike | | Thamnophilidae | | Aracruz | |  |  |
| THAMB10 | *Plasmodium* | *Thamnophilus ambiguus* | Sooretama Slaty-Antshrike | | Thamnophilidae | | Aracruz | | JX029896 | |
| THCAE01 | *Plasmodium* | *Sakesphorus cristatus* (3) | Silvery-cheeked Antshrike | | Thamnophilidae | | Bocaiúva | | JX021457 | |
|  |  | *Sakesphorus cristatus* | Silvery-cheeked Antshrike | | Thamnophilidae | | Manga | |  |  |
|  |  | *Thamnophilus ambiguus* | Sooretama Slaty-Antshrike | | Thamnophilidae | | Bocaiúva | |  |  |
|  |  | *Thamnophilus caerulescens* (3) | Variable Antshrike | | Thamnophilidae | | Nova Lima | |  |  |
| THSAY01 | *Plasmodium* | *Thraupis sayaca* (2) | Sayaca Tanager | | Thraupidae | | Bocaiúva | | JX029891 | |
|  |  | *Thlypopsis sordida* | Orange-headed Tanager | | Thraupidae | | Bocaiúva | |  |  |
| THSAY02 | *Plasmodium* | *Thraupis sayaca* (3) | Sayaca Tanager | | Thraupidae | | Aracruz | | JX029898 | |
|  |  | *Trichothraupis melanops* | Black-goggled Tanager | | Thraupidae | | Caratinga | |  |  |
| TOFLA01 | *Plasmodium* | *Phaeomyias murina* | Mouse-colored Tyrannulet | | Tyrannidae | | Felixlândia | | JX021472 | |
|  |  | *Tolmomyias flaviventris* (8) | Yellow-breasted Flycatcher | | Tyrannidae | | Jequitinhonha | |  |  |
|  |  | *Tolmomyias flaviventris* (2) | Yellow-breasted Flycatcher | | Tyrannidae | | Salto da Divisa | |  |  |
|  |  | *Tolmomyias flaviventris* | Yellow-breasted Flycatcher | | Tyrannidae | | Manga | |  |  |
| TOFLA02 | *Haemoproteus* | *Myiarchus tyrannulus* | Brown-crested Flycatcher | | Tyrannidae | | Salto da Divisa | | JX029908 | |
|  |  | *Tolmomyias flaviventris* | Yellow-breasted Flycatcher | | Tyrannidae | | Jequitinhonha | |  |  |
| TOFLA03 | *Haemoproteus* | *Tolmomyias flaviventris* (2) | Yellow-breasted Flycatcher | | Tyrannidae | | Aracruz | | JX029916 | |
| TOSUL01 | *Haemoproteus* | *Tolmomyias sulphurescens* | Yellow-olive Flycatcher | | Tyrannidae | | B. de Minas | | JX029915 | |
|  |  | *Tolmomyias sulphurescens* | Yellow-olive Flycatcher | | Tyrannidae | | Felixlândia | |  |  |
| TRMEL01 | *Plasmodium* | *Trichothraupis melanops* | Black-goggled Tanager | | Thraupidae | | Caratinga | | JX021467 | |
| TRMEL02 | *Plasmodium* | *Anabazenops fuscus* | White-collared Foliage-gleaner | | Furnariidae | | Caratinga | | JX021468 | |
|  |  | *Trichothraupis melanops* (2) | Yellow-olive Flycatcher | | Thraupidae | | Caratinga | |  |  |
|  |  | *Vireo olivaceus* | Red-eyed Vireo | | Vireonidae | | Aracruz | |  |  |
|  |  | *Zonotrichia capensis* | Rufous-collared Sparrow | | Emberizidae | | Aracruz | |  |  |
| TRMUS01 | *Haemoproteus* | *Troglodytes musculus* (2) | Southern House Wren | | Troglodytidae | | Aracruz | | JX029918 | |
| TUAMA01 | *Plasmodium* | *Turdus albicollis* (2) | White-necked Thrush | | Turdidae | | Bocaiúva | | JX021477 | |
|  |  | *Turdus amaurochalinus* | Creamy-bellied Thrush | | Turdidae | | Salto da Divisa | |  |  |
|  |  | *Turdus amaurochalinus* | Creamy-bellied Thrush | | Turdidae | | Bocaiúva | |  |  |
|  |  | *Turdus amaurochalinus* | Creamy-bellied Thrush | | Turdidae | | Jequitinhonha | |  |  |
|  |  | *Turdus amaurochalinus* | Creamy-bellied Thrush | | Turdidae | | Manga | |  |  |
|  |  | *Turdus leucomelas* | Pale-breasted Thrush | | Turdidae | | Aracruz | |  |  |
| TULEU01 | *Plasmodium* | *Thamnophilus ambiguus* | Sooretama Slaty-Antshrike | | Thamnophilidae | | Salto da Divisa | | JX021460 | |
|  |  | *Turdus amaurochalinus* | Creamy-bellied Thrush | | Turdidae | | Jequitinhonha | |  |  |
|  |  | *Turdus leucomelas* | Pale-breasted Thrush | | Turdidae | | Caratinga | |  |  |
|  |  | *Turdus leucomelas* (4) | Pale-breasted Thrush | | Turdidae | | Felixlândia | |  |  |
|  |  | *Turdus leucomelas* | Pale-breasted Thrush | | Turdidae | | Jequitinhonha | |  |  |
|  |  | *Turdus leucomelas* (2) | Pale-breasted Thrush | | Turdidae | | B. de Minas | |  |  |
|  |  | *Tyrannus melancholicus* (2) | Tropical Kingbird | | Tyrannidae | | Felixlândia | |  |  |
| TULEU02 | *Plasmodium* | *Coryphospingus pileatus* | Grey Pileated Finch | | Emberizidae | | Jequitinhonha | | JX021466 | |
|  |  | *Turdus leucomelas* | Pale-breasted Thrush | | Turdidae | | Caratinga | |  |  |
|  |  | *Turdus leucomelas* | Pale-breasted Thrush | | Turdidae | | Felixlândia | |  |  |
| TULEU03 | *Plasmodium* | *Turdus leucomelas* (3) | Pale-breasted Thrush | | Turdidae | | Felixlândia | | JX029871 | |
| TULEU04 | *Plasmodium* | *Turdus leucomelas* (2) | Pale-breasted Thrush | | Turdidae | | Felixlândia | | JX029872 | |
| TULEU05 | *Plasmodium* | *Turdus leucomelas* | Pale-breasted Thrush | | Turdidae | | Felixlândia | | JX029874 | |
| TULEU06 | *Plasmodium* | *Pachyramphus viridis* | Green-backed Becard | | Tyrannidae | | B. de Minas | | JX029878 | |
|  |  | *Turdus leucomelas* | Pale-breasted Thrush | | Turdidae | | Jequitinhonha | |  |  |
|  |  | *Turdus leucomelas* (2) | Pale-breasted Thrush | | Turdidae | | Bocaiúva | |  |  |
| TULEU07 | *Plasmodium* | *Turdus amaurochalinus* | Creamy-bellied Thrush | | Turdidae | | Felixlândia | | JX029881 | |
|  |  | *Turdus leucomelas* | Pale-breasted Thrush | | Turdidae | | Felixlândia | |  |  |
| TULEU08 | *Plasmodium* | *Turdus leucomelas* | Pale-breasted Thrush | | Turdidae | | Jequitinhonha | | JX029886 | |
| TUMIG03 | *Plasmodium* | *Turdus amaurochalinus* | Creamy-bellied Thrush | | Turdidae | | Nova Lima | | JX021462 | |
|  |  | *Turdus amaurochalinus* (4) | Creamy-bellied Thrush | | Turdidae | | Felixlândia | |  |  |
|  |  | *Turdus amaurochalinus* | Creamy-bellied Thrush | | Turdidae | | Bocaiúva | |  |  |
| TURUF01 | *Plasmodium* | *Turdus rufiventris* | Rufous-bellied Thrush | | Turdidae | | Nova Lima | | JX021473 | |
|  |  | *Turdus rufiventris* (2) | Rufous-bellied Thrush | | Turdidae | | Caratinga | |  |  |
| TURUF02 | *Haemoproteus* | *Turdus rufiventris* | Rufous-bellied Thrush | | Turdidae | | Caratinga | | JX029920 | |
| VIOLI01 | *Plasmodium* | *Troglodytes musculus* | Southern House Wren | | Troglodytidae | | Aracruz | | JX029897 | |
|  |  | *Vireo olivaceus* (6) | Red-eyed Vireo | | Vireonidae | | Aracruz | |  |  |

*Number of infected individuals per site is indicated in parenthesis
